# Asiatic Cholera and Cholera Morbus

**Published:** 1857-11

**Authors:** Th. Polland


*Asiatic Cholera, and Cholera Morbus.* By Th. Polland, M. D.

We believe Asiatic cholera to be cholera morbus in *epidemic* form; and it may not be inappropriate at this season to give some reasons drawn from analogy, and facts for this belief. We are aware that many, possibly a majority of the profession do not admit the diseases to be the same, and look upon Asiatic cholera "sui generis." We are the more induced to say something on the subject at this time, as we have been hearing of sporadic cases of *Asiatic* cholera; and as we hear of them every summer from men of high standing and authority, and whose opinions have weight, both among the medical and non-medical public, whose fears are aroused, and whose anxiety about personal safety and injury to business is not inconsiderable. This belief, so common in the prosession, we are convinced has arisen from want of reflection, and we have been rather astonished at the idea that epidemic or Asiatic cholera is a new disease, originating about 1817. That this affection was first apparent in Europe about 1830, is a matter of history. Why it never spread from the east to the west before this time, we know not. Possibly the increasing commerce of the world had some agency; but as this might involve the doctrine of contagion, we do not design to express any opinion to this effect, and merely mention the fact.

That cholera morbus existed in an epidemic form at an early age, is plainly evident. It is described by Aretæus and Paulus Ægineta,* and by Cœlius Auræliauus, the latter of whom observes it is "a torment of the bowels, looseness and oppression of the præcordia, vomiting of humor and yellow bile, succeeded by matter somewhat resembling the white of an egg; also great heat, insatiable thirst, tension of the sinews and calves of the legs and arms; præcordia drawn upwards, with pain like that of the iliac passion—the countenance being collapsed, and the eyes red."

Bontius, a Dutch physician, writing in 1629, says, in describing *cholera morbus* at Batavia, "It is extremely frequent. In the cholera, hot billious matter irritating the stomach and intestines is incessantly and copiously discharged by the mouth and anus. It is a disorder of the most acute kind, and therefore requires immediate application. * * * *Those who are seized with the desorder generally die, and that so quickly as in the space of 24 hours at most.*"

He also mentions the weak pulse, difficult respiration, coldness of surface, restlessness, cold sweat, also spasm, which he says exists much less than in the Indies, and cites on case of the steward of the hospital in his country who died of this disease, with convulsions, in six hours. Though he does not say the disease was epidemic cholera, he has evidently and truly portrayed that disease.

Sydenham describes an epidemic cholera morbus in 1629 as follows:

---

*Vid. Bell and Condie on Cholera, p. 143.

"Immoderate vomiting, and a discharge of vitiated humors by stool, with great difficulty and pain; violent pain, and distension of the abdomen and intestines; heart-burn, thirst, quick pulse, heat and anxiety, and frequently a small and irregular pulse; great nausea, and sometimes colliquative sweats; contraction of the limbs; fainting, coldness of the extremities, and other like symptoms, which greatly terrify the attendants, and often destroy the patient in 24 hours."

And again, in a letter to Dr. Brady, he says that "at the close of the summer (1676) the cholera morbus raged epidemically, and being rendered more severe by the extraordinary heat of the season, was accompanied with more violent and inveterate convulsions than I had hitherto observed; for not only the abdomen but all the muscles of the body, and especially those of the arms and legs were effected with terrible spasms."

Dr. Paisley, who wrote in 1774, speaking of cholera morbus, says, "It is often epidemic among the blacks (natives,) whom it destroys quickly, as their relaxed habits cannot support the effects of sudden evacuations nor the more powerful operation of diseased bile." He also says, "When it is epidemic here it is totally a disease of highly putrid bile, which operates on the system *as a poison, and brings on sudden prostration of strength, and spasms over the whole surface of the body.*" These latter remarks refer to the years 1769-70.

Sonnerat, who traveled in India in 1774 to 1781, says, "There is another epidemical disorder which reigns, and in 24 hours or less carries off those it attacks. The symptoms of the disease were a watery flux, accompanied with vomiting and extreme faintness, a burning thurst and oppression of the breast, and a *suppression of urine.* They were often without pulse, the hands and ears were cold, the face lengthened, and eyes sunken."

He also describes spasms and colicky pains as being attendants on this affection, which was evidently epidemic cholera.

British army officers mention a similar disease which prevailed in 1775, at Mauritius, and in 1781, Mr. Jameson, secretary to the Calcutta medical board, says, "A disease resembling cholera attacked a division of Bengal troops of 5,000 men, with inconceivable fury. Men dropped down by dozens, with dreadful spasms of the extremities and trunk, and vomiting and purging, and some died in an hour."

Curtis, surgeon in the English navy at Madras, says, the "mort de chien, or cramp, had been very frequently and fatal among the seament the sickness being attended with vomiting, purging, coldness of extremities, sinking and lividity of the face, the eyes sunken, the pulse scarcely, or not at all perceptible, great thirst, soaken condition of the hands, cramp, thin, watery, mucous discharges from the bowels, spasms, but with clear and undisturbed intellect to the last."

The "Bengal report" states, that in April 1783 cholera destroyed 20,000 people, on an occasion of a festival.

Dr. Duffin of the English army in India, says, "In 1884, cholera morbus rages with great violence, and is so rapid in its progress that many of the men are carried off in twelve hours. They are seized with nausea, frequent heats and chills, then cold sweats, severe griping and purging of *bilious* colluvies."

Dr. Davis, describing the same epidemic, speaks "of the bladder being empty and contracted," and Mr. Thompson mentions the same thing.

Dr Jas. Johnson, in his work on Tropical Climates, describes cholera in the east in 1804; and after giving the history of a case, says "This may serve as a specimen of that dreadful disease which has obtained the appellation of 'mort de chien' or death of the dog."

Other observers in the east have described this affection as prevailing in 1790 and 1814, calling it "epidemic cholera morbus," "cholera," and "cholera asphyxia"—the name of *Asiatic* cholera being first given to the epidemic which started in the east in 1817, traveling west—another instance of a *new* name misleading, and including the belief of a *new* disease.

Apart from fact, analogy supports the belief of the identity of the two diseases. All observers are aware that epidemic and sporadic disentary differ considerably in their intensity, in some of their symptoms, and in prognosis—sporadic dysentery in our climate being rarely fatal; epidemic dysentery frequently so. So of erysipelas—in its sporadic form its tendency is to recovery—in its epidemic character it is often fatal, particularly in hospitals, and in that form called "black tongue," and in that variety attacking the peritoneum, for it is well ascertained that when the disease is epidemic, it is apt to be communicated by nurses and physicians, and probably through the atmosphere, to lying in women, constituting a fatal form of puerperal fever. And these remarks apply generally to epidemic disease, which is almost always more severe than when sporadic, and always assuming new appearances and symptoms.

Several points of diagnosis between cholera morbus and Asiatic cholera have been laid down. Among them it has been stated by Watson and others, that in the latter the discharges show an evidence of want of secretion of bile, while in the former they are billious. I am sure every one's observation will disprove this assertion. In mild cases only of cholera morbus a copious secretion and discharge of bile speedily occurs, and stops the disease, while in all violent cases there is almost always an entire absence of bile in the evacuations. The older writers describe common cholera as being generally attended with absence of bilious discharges; and on the other hand, some of these writers, as already quoted, speak of the subjects of epidemic cholera morbus (evidently our Asiatic cholera of modern times) as "being overcome by the more powerful operation of diseased bile," thus rendering it probable that there is no invariable rule on this point, though the general rule, as we have stated, is that in violent cases of this disease there is absence of bile in the secretions.

Another point of difference is stated to be "suppression of urine" in Asiatic cholera. Now it is evident that this is a symptom dependent on prostration and want of innervation, and may occur as well in fatal cases of sporadic cholera as of Asiatic. Again we are told the convalescence is tedious in one affection, and rapid in the other, and that secondary fever follows only Asiatic cholera. We admit Asiatic cholera is attendant with more prostration, graver symptoms and longer collapse than in sporadic cases, and the longer an organ is congested, the more probability there is of infl mn a'ion and consequent secondary f ver.

Another point of distinction is said to be that cholera morbus is brought on by imprudence in eating, and Asiatic cholera is not. Wh re a disease is epidemic, and the whole atmosphere loaded with poisonous material, it of course requires less exciting causes to develop the malady than when the atmosphere is healthy. It is, however, a matter of history that the badly nourished, badly arid and imprudent portion of the people are those who are subject to Asiatic cholera. A man living in a well ventilated, dry apartment, who is temperate in eating and drinking, and regular in hours need not fear cholera, even though he live in the impure atmosphere of a diseased city. Possibly cholera may sometimes affect such a person, and we sometimes hear of men in the better walks of life being its victims, but we believe if the history of these individuals could be known, it would be found that they had committed some imprudence in the use of diet or alcoholic drinks, or lost rest, or in some way impaired the strength and tone of the digestive apparatus; for imprudent drinking particularly will do this as effectually as improper eating.

And again—some, hard pressed for differential diagnosis, tell us that cholera morbus is rarely fatal. This is true; but this is true of all sporadic disease compared with epidemic. Cholera morbus too, including cholera infantum, is more fatal than some are willing to admit. In Philadelphia, in ten years ending January 1, 1832, from the report of the board of health, the deaths from sporadic cholera were 2,437, of which 114

were over ten years of age, and this too at a time when the population was small as compared with the present.

Those who dwell on the rare fatality of cholera morbus, are, we suspect, those who, seeing a very severe case of sporadic disease, pronounce it Asiatic cholera, because they discover here, particularly if the patient is moribund, all the symptoms they ascribe to Asiatic cholera.

We design to say a few words of treatment, which, reasoning from analogy of the most successful treatment in sporadic cholera, and from facts and statistics, we believe to be *opium*. We would not neglect other remedies and adjuvants, calomel, counter-irritants and stimulants, but we look on this one medicine as the sheet-anchor, and we have often asked the question, with what other remedy can you restrain the vomiting and purging to prevent collapse, and give your calomel an opportunity of acting on the liver? and have never obtained a satisfactory reply. We have been told that in some caces there is death without vomiting and purging.—These caces are very rare, and form the exception, all observers and authors agreeing upon the general fact that diarrhœa and vomiting are the precursory symptoms of Asiatic cholera; and in cases of death without vomiting and purging, post-mortem examination has proved the intestines are loaded with effused serum, which probably was not thrown off per anum from want of muscular power, the poison having produced sudden sedation and prostration of the whole nervous system. Possibly opium given early in these would have allayed the irritation of the mucous membrane, have produced regular distribution of nerve power and given time for remedies, and the "vis medicatrix" to have had fair play.

The writer of this has seen cholera supervene while the system was fully under the influence of mercury; and we are much disposed to coincide with those who have expressed the opinion that the secretion and discharge of bile and return of the secretions is rather the *effect* than the *case* of the system righting itself, and that the "post hoc, propter hoc" reasoning has been applied to the action of calomel in cholera.

We have never been a believer in the *congestive* action of opium in cholera. By subduing neuro-sthenia and equalizing irregular distribution of nerve force, we believe it is decidedly *anticongestive* in this as well as other affections. Improperly used in checking secretion, and producing constipation of the bowels, it may prove congestive to the brain and other organs, but this cannot apply to cholera.

In this connection we may properly allude to the statistics furnished by the "report of the London board of health,"* to which are attached

*Vide Va. Med. and Surg. Journal, July 1855, p. 39.

the distinguished names of Drs. Pain, Alderson, Babingson and Tweedie. 2,749 well developed cases of cholera are reported, the general per centage of death following each plan of treatment being of

| | | |
|---|---|---|
| Eliminants, - - - - - · | 71.7 | per cent, |
| Stimulants, - - - - - | 54. | " |
| Alteratives (calomel and opium,) - - - | 36.2 | " |
| Astringents (chalk and opium,) - - - | 20,3 | " |

It is proper to state that of the 150 cases treated with the chalk and opium with such favorable results, not one-third had entered the stage of collapse, while of the 166 treated by salines, three-quarters were collapsed ; and of the 124 cases in which caster oil treatment was adopted, all but 12 were in collapse. Still this testimony is very strong in favor of the opium treatment.

The fairest test is to take the collapse cases, and draw the comparison, the fatality being as follows :

| | | |
|---|---|---|
| Calomel and opium, - - - - | 59 | per cent. |
| Calomel (large doses,) - - - - | 60 | " |
| Salines, - - - - | 62 | " |
| Chalk and opium, - - - - | 63 | " |
| Calomel (small doses,) - - - - | 73 | " |
| Caster oil, - - - - - | 75 | " |
| Sulphuric acid, - - - - - | 78 | " |

Here, though collapse had taken place, the opium with calomel was more successful than calomel alone ; and it is in this state that opium has been so much condemned, and regarded by some, as fatal. Before collapse the opium treatment was, as evidenced by the first table, by far most successful.

Ten thousand coses of cholera diarrhœa were treated ; and the committee say the sulphuric acid treatment was the most efficacious ever practiced—the saline treatment being least successful—and the calomel and opium next in efficacy to the sulphuric acid.

In a disease, necessarily so fatal as cholera, when fully formed, diversity of opinion will probably continue to exist as regards treatment ; but we conceive that every one who will fairly investigate the snbject, must come to the conclusion we have endeavored to enforce, that cholera morbus and Asiatic cholera are one and the same disease. Analogy, the fact that the same organs are affected in the so-called two diseases in the same way, and the additional consideration that a person dying with sporadic and another with Asiatic cholera, exhibit, as far as we can learn, the same condition of things, are all, we conceive, very strong evidences of the truth of our position.

If we succeed at all in allaying the constant talk every summer about "Asiatic cholera," we shall be paid for our trouble in prepairing this paper.—*Va. Med. Journal.*

